# Supplementary material for: Social subordination is associated with better cognitive performance and higher theta coherence of the mPFC-vHPC circuit in male rats
Source: PLoS One. 2025 Apr 16;20(4):e0320952. doi: 10.1371/journal.pone.0320952 (PMC12002502; doi:10.1371/journal.pone.0320952)
Supplement: Attachment — Submitted filename: Respnse to reviewer_ 1403_9_14.docx [file pone.0320952.s002.docx]

**#Journal Requirements:**

1.When submitting your revision, we need you to address these additional requirements

**Answer: Thanks for your comments, the article has been changed to the requested format.**

2. To comply with PLOS ONE submissions requirements, in your Methods section, please provide additional information regarding the experiments involving animals and ensure you have included details on (1) methods of sacrifice, (2) methods of anesthesia and/or analgesia, and (3) efforts to alleviate suffering.

**Answer: Thanks for your comment, all points have been addressed and highlighted in the manuscript.**

\#**method: To implant the electrodes in the specified areas, at first the rat was anesthetized using a mixture of ketamine (100 mg/kg) and xylazine (10 mg/ kg). Then, the anesthetized rat was placed in the stereotaxic apparatus (Toosbioresearch, Mashhad, Iran) and a heating pad was used to maintain the rat body temperature at 37 °C during surgery. Anesthesia depth was checked by tail and foot pinch reflexes. Vitamin A ointment was also used to prevent the animal's eyes from drying out during surgery.**

**To ensure accurate electrode placement, rats were first deeply anesthetized with** **carbon dioxide and after complete cessation of breathing and ocular pallor the animals were rapidly decapitated by guillotine and then the brains of the rats were carefully removed and placed in 4% paraformaldehyde at 4°C for 48h. Then, using a vibroslicer, the brains were cut and stained with methylene blue and examined under a microscope (AC 230V 50 Hz, Fig 1b).**

.3. We note that the grant information you provided in the ‘Funding Information’ and ‘Financial Disclosure’ sections do not match. When you resubmit, please ensure that you provide the correct grant numbers for the awards you received for your study in the ‘Funding Information’ section.

**Answer: Thank you for notifying us about this matter, we considered this point during resubmission.**

4. Thank you for stating the following financial disclosure: "We would thank the Iran National Science Foundation for foundation support (grant number: 4012752)." Please state what role the funders took in the study. If the funders had no role, please state: "The funders had no role in study design, data collection and analysis, decision to publish, or preparation of the manuscript."

**Answer:** **The funders had no role in study design, data collection and analysis, decision to publish, or preparation of the manuscript.**

5. Thank you for stating the following in the Acknowledgments Section of your manuscript: "We would thank the Iran National Science Foundation for foundation support (grant number: 4012752)".

We note that you have provided funding information that is not currently declared in your Funding Statement. However, funding information should not appear in the Acknowledgments section or other areas of your manuscript. We will only publish funding information present in the Funding Statement section of the online submission form. Please remove any funding-related text from the manuscript and let us know how you would like to update your Funding Statement. Currently, your Funding Statement reads as follows: "We would thank the Iran National Science Foundation for foundation support (grant number: 4012752)." Please include your amended statements within your cover letter; we will change the online submission form on your behalf.

**Answer: Thanks for mentioning this point, the mentioned part was deleted from the manuscript and was added to cover letter.**

6. In the online submission form, you indicated that your data is available only on request from a third party. Please note that your Data Availability Statement is currently missing the contact details for the third party, such as an email address or a link to where data requests can be made. Please update your statement with the missing information.

**Answer: We are sorry for the inconvenience. Thank you for reminding us.**

**Data Availability:** The data linked to this research is openly accessible on the Open Science Framework (OSF) platform. To access and download the dataset, please follow this link:

[**https://osf.io/sjex3/?view_only=c0da26cdab6e4141995620a703a016d8**](https://osf.io/sjex3/?view_only=c0da26cdab6e4141995620a703a016d8)

**#Reviewer1**

1. The authors investigated the relationship between social dominance hierarchies and neural connections between the medial prefrontal cortex (mPFC) and the ventral hippocampus (vHPC). The authors then addressed the role of social hierarchy and spatial working memory (SWM). Although it is a very interesting research topic, the vast amount of data and lengthy papers often confuse readers. This paper contains a lot of information that is already known, and it is important to cite previous research and note any discrepancies between the authors' results and those of other studies. What were the new findings in the study conducted by the authors? Also, what has already been clarified? Please organize these. We would like you to revise your paper with consideration to summarizing it concisely. findings from the study and to summarize explanations of non-significant data concisely, limiting interpretations to those that are specifically needed to support the discussion**.**

**Answer: Thank you very much for pointing these out, the text of the manuscript were rearranged and presented in the following manner as was stated by the esteemed reviewer. The changes are highlighted in the revised manuscript text.**

**#Abstract: Electrophysiological data revealed significant theta correlation and coherence of the mPFC-vHPC circuit that was higher in subordinates. By increasing task difficulty, theta rhythm coherency was reduced for all social ranks but subordinates maintained better task performance with less reduction of theta coherence.** **These findings are presented for the first time and underscore the association between different social hierarchies and working memory performance within the mPFC-vHPC circuit; highlighting the influence of social rank on implicated circuit.**

**#Introduction:** **Considering controversial results from different studies regarding relationship between social hierarchy and memory performance, our study tries for the first time to elucidate the impact of social hierarchy on SWM during two degrees of task difficulty, specifically aiming at shedding light on the underlying functional connectivity within the mPFC-vHPC circuit by LFP recording in sibling male rats with different social ranks.**

**#Results: In this section, an attempt was made to omit parts of the results that were non- significant and were indicated with track changes in the text.**

**#Discussion:** **This study** **investigated the impact of social hierarchy on SWM performance in different social statuses of male rats, focusing on the mPFC-vHPC circuit for the first time, a common neural circuit implicated in both social hierarchy and SWM.**

**According to our findings, in this study, the subordinate rats showed better performance at the behavioral and electrophysiological levels compared to the other two groups. At the behavioral level, the subordinate rats were able to respond faster in choosing the target arm, as well as have a higher response accuracy, and at the level of electrophysiology, they had a higher theta correlation and coherence of mPFC-vHPC than the other two ranks. As the task becomes more difficult, the behavioral performance of the dominant rats remains unchanged, while the subordinate rats experience a decrease in performance. At the level of electrophysiology, in all three social groups, the coherence of the circuit decreases with the increases in the difficulty of the task. This shows that the dominant rats were able to maintain the same behavioral performance despite reduced mPFC-vHPC coherency.**

2. In addition, in terms of materials and methods, the main conditions should be those that allow the experiment to be reproduced with good reproducibility, and it is appropriate to include the numerical values etc. required for the experiment. The T-maze is a widely used method in behavioral analysis, so explanations of it will be kept to a minimum; more important are details about lighting, time of measurement, etc. General methods should be described by simply saying "according to standard methods," and if the authors' original methods or measurement conditions are special, these should be described.

**Answer: Thank you for this point, we have provided more details about situation of conducting T-maze protocol.**

**#method: The rats were obtained from Urmia Medical Sciences University and housed at 21±2 °C, 12:12 light-dark** **cycle (from 8 am to 8 pm). They were kept in standard animal research facilities in which food and water were freely available.**

**According to the standard protocol for T-maze running [23], we used the spontaneous alternation which is performed without a food reward and habituation. Here, it is the novelty of the maze arms that drives the spontaneous exploration. We used this protocol because of rats’ difference in desire for food between dominant and subordinate rats [27]. In T-maze task, for the sample phase, each rat was first placed at the starting point (i.e., the base of the arm perpendicular to the left and right arms) of the maze and was let to choose between left or right arms. After the sample phase, the rat was locked in the selected arm for 30 seconds and then returned to the starting point. At the starting point, depending on the difficulty of the task, rats were locked at that point for 30 s (easy) or 5 min (difficult), a delay time between sample and choice run trials. In this protocol, it is possible to freely choose the target arm in both the sample phase and choice phase [23]. The difficulty of the task was alternated every day for eight days (four days of 30 seconds difficulty and four days of 5 minutes). Six trials were performed every day (one sample trial and five choice trials). The time limit for the rats to remain in the maze was 180 seconds [23]. All movements of the animals were recorded via a video camera located above the maze for subsequent analysis. All tests were conducted in the afternoon every day from 3 pm to 6 pm. Also, the light intensity during the experiment was 100 lux.**

3. The results section is often filled with data stating that no significant differences were observed. If data is particularly necessary, it is necessary to include it, but if it is not, and can be understood by looking at figures, etc., it may be possible to omit it. Although it is a minor point, the way of writing p-values should be standardized (p<0.001 or p<.001). Also, significant figures should be considered. Regarding the writing style of papers, figure legends are currently written in the main text of papers, but it is common to put them together at the end of the main text.

**Answer: Thanks for your comments, all of the changes have been applied carefully. Some of the non-significant data were particularly necessary, so they remained but some of them weren’t necessary and they have been omitted and indicated with track changes in the manuscript. In the manuscript, all the changes have been highlighted in the Results section. Also, we have written the p-values in standardized form (p<.001). All figure legends are put together at the end of the main text.**

4. Overall, it is not clear in reviewing this study what is novel. The experiments in this study included pilot and follow-up experiments, and ideally the main data should be those experiments that focused on the purpose of the study. The paper should cite references to what has already been published in previous research papers, mention what the authors have newly clarified in this study, and focus on the methodology used to arrive at this conclusion and the data necessary for discussion.

**Answer: Sorry to cause confusion, according to the answer given to the question one, the changes in introduction are as followed “****Considering controversial results from different studies regarding relationship between social hierarchy and memory performance, our study tries for the first time to elucidate the impact of social hierarchy on SWM during two degrees of task difficulty, specifically aiming at shedding light on the underlying functional connectivity within the mPFC-vHPC circuit by LFP recording in sibling male rats with different social ranks. Therefore, according to the literature of research, which were mainly behavioral studies, and in some of them the subordinate group performed better [10] and in others the dominant group [21,22], our study tried for the first time to compare the performance of groups with different social levels in neural circuits (mPFC-vHPC) and LFP recording in two different degrees of difficulty.”**

**Discussion “According to our findings, in this study, the subordinate rats showed better performance at the behavioral and electrophysiological levels compared to the other two groups. At the behavioral level, the subordinate rats were able to respond faster in choosing the target arm, as well as have a higher response accuracy, and at the level of electrophysiology, they had a higher theta correlation and coherence of mPFC-vHPC than the other two ranks. As the task becomes more difficult, the behavioral performance of the dominant rats remains unchanged, while the subordinate rats experience a decrease in performance. At the level of electrophysiology, in all three social groups, the coherence of the circuit decreases with the increases in the difficulty of the task. This shows that the dominant rats were able to maintain the same behavioral performance despite reduced mPFC-vHPC coherency”.**

**#Reviewer2**

1. Given the dHPC's primary role in spatial working memory, alongside the vHPC, the authors should provide a more detailed explanation for their decision to focus solely on the ventral part of this brain region.

**Answer:** **Although most studies have investigated the synchronization between PFC and dHPC,** **the synchronization between PFC and vHPC is stronger. For instance, O'Neill et al.'s study (2014) on rats showed that the input power of vHPC is very important for mPFC and plays a significant role in synchronizing the oscillatory activity of mPFC with dHPC area. In other words, it is the theta rhythm in the vHPC that regulates the synchrony between the other two regions [1,2]. Moreover, regarding working memory performance, the synchronization of vHPC-mPFC circuit plays a determining role.**

**In the manuscript changes are as follows.**

**#Introduction: Moreover, the intricate interplay between the mPFC and vHPC extends to their joint involvement in SWM tasks. The successful execution of SWM tasks relies on the coordinated activity of these regions, with the ventral hippocampus (vHPC) and dorsal hippocampus (dHPC) contributing to spatial encoding, and the mPFC facilitating executive control [16]. Notably, the synchronization of theta rhythms between the mPFC and vHPC fosters long-range connectivity crucial for SWM formation [17,18]. In this study, we used vHPC because the synchronization between PFC and vHPC is stronger than dHPC. Also, inputs from vHPC are very important for mPFC and plays a significant role in synchronizing the oscillatory activity of mPFC with dHPC area. In other words, theta rhythm in the vHPC regulates the synchrony between these two regions [19,20].**

2. A more detailed explanation of the system used to determine the precise timing of the decision point is necessary. Did they use video-EEG?

**Answer:** **In order to accurately extract the decision point time, videos were taken from all recorded moments and the times were extracted from videos. We described the details in data analysis section in method. We didn’t use video-EEG.**

**#Method:** **The process of extracting the data from the electrophysiology section was done by using videos recorded during the LFP signal recording, in order to match the time of behavioral activity with LFP signals. Then, the videos were analyzed and the decision point was extracted with millisecond precision. This time is actually the moment of turning the rat’s head towards the target for the last time, which three seconds before this time and one second after this moment (4 seconds in total) was considered as the decision point.**

3. a) I have some questions about the T-maze task. Could the authors please provide a more detailed description of the protocol? Did they impose a time limit for the animals to complete the task? Were any animals excluded from the behavioral testing?"

**Answer: we thank the esteemed reviewer for making all of these points, all of them have been addressed. The time limit for the rats to remain in the maze was 180 seconds. We didn’t exclude any animals from the behavioral testing.**

**#Method: About T-maze “****According to the standard protocol for T-maze running [23], we used the spontaneous alternation which is performed without a food reward and habituation. Here, it is the novelty of the maze arms that drives the spontaneous exploration. We used this protocol because of rats’ difference in desire for food between dominant and subordinate rats [27]. In T-maze task, for the sample phase, each rat was first placed at the starting point (i.e., the base of the arm perpendicular to the left and right arms) of the maze and was let to choose between left or right arms. After the sample phase, the rat was locked in the selected arm for 30 seconds and then returned to the starting point. At the starting point, depending on the difficulty of the task, rats were locked at that point for 30 s (easy) or 5 min (difficult), a delay time between sample and choice run trials. In this protocol, it is possible to freely choose the target arm in both the sample phase and choice phase [23]. The difficulty of the task was alternated every day for eight days (four days of 30 seconds difficulty and four days of 5 minutes). Six trials were performed every day (one sample trial and five choice trials). The time limit for the rats to remain in the maze was 180 seconds [23]. All movements of the animals were recorded via a video camera located above the maze for subsequent analysis. All tests were conducted in the afternoon every day from 3 pm to 6 pm. Also, the light intensity during the experiment was 100 lux.**

b) Do the T-maze results represent the cumulative number of correct and incorrect responses across the eight days of trials? This information should be clarified in the Methods or Figure legend. Additionally, were LFP analyses conducted for each individual trial?

**Answer: The T-maze** **results represent the cumulative number of correct and incorrect responses across the four days of trials; because the difficulty of the task was alternated every day for eight days (four days of 30 seconds difficulty and four days of 5 minutes). Six trials were performed every day (one sample trial and five choice trials). Also, LFP analyses conducted for each individual trial.**

**In data analysis section also, we have addressed the mentioned questions as follows, “Then these files were converted into readable files for MATLAB version 2022b (MathWorks, Natick, MA, USA), and the desired times were extracted from the recorded signals. In this way, theta distribution power, theta correlation between mPFC and vHPC regions, and theta coherence between these two regions were extracted in the frequency range of 4-12 Hz at the decision point of the rat to choose the desired arm. The desired variables of this research were analyzed using functions available in MATLAB such as “mscohere” function for circuit coherence. Results represent the cumulative number of correct and incorrect responses across the four days of trials; because the difficulty of the task was alternated every day for eight days (four days of 30 seconds difficulty and four days of 5 minutes difficulty). Also, LFP analyses conducted for each individual trial.**

c) Given that mice exhibited decreased speed and increased latency over successive trials, did the authors observe any variations in performance within a single day or across the eight-day testing period?

**Answer: About the last part of the questions, we observed variations during four-day testing period in both of task difficulties. We have prepared day by day figure regarding variations in performance for both of latency and correct responses [Fig 1]. Also, we have prepared trial by trial figures that indicated every social group performance from trial one to trial five in day one and day four of LFP recording. Because there were many pictures, we used day one and day four for indicating average performance of rats through the trials [Fig 2, Fig3]. According to the results subordinate group showed decreased latency through day one to day four and also through trial one to trial five and increased correct response through day one to day four.**

**Fig 1. Day by day analysis of percentage of correct responses (a) and latency of correct choice (b) in easy (30 s) and difficult (5 min) tasks.**

**Fig 2. Trial by trial analysis of latency to choose correct arm in easy task in day 1 and day 4 of LFP recording.** a) Trial by trial latency in dominant group. b) Trial by trial latency in middle-ranked group. c) Trial by trial latency in subordinate group.

**Fig 3. Trial by trial analysis of latency to choose correct arm in difficult task in day 1 and day 4 of LFP recording.** a) Trial by trial latency in in dominant group. b) Trial by trial latency in middle-ranked group. c) Trial by trial latency in subordinate group.

4. Regarding Figure 1, authors should improve the pictures displaying the brain section in which the electrodes were implanted and describe how did they acquired the image.

**Answer: Thanks for your comment, the mentioned item has been corrected in the text and the resolution of the images has been increased [Fig 4].**

\#**Method: To ensure accurate electrode placement, rats were first deeply anesthetized with carbon dioxide and then the brains of the rats were carefully removed and placed in 4% paraformaldehyde at 4°C for 48h. Then, using a vibroslicer, the brains were cut and stained with methylene blue and examined under a microscope (AC 230V 50 Hz, Fig 1b).**

Fig4. **Timeline of behavioral and electrophysiological procedures**. a) Different phases of social development of three natal littermates before and after tube test: Dominant (D, red), Middle-ranked (M, gray), and Subordinate (S, blue) b) Timeline of surgery, T-maze task, LFP recording in decision point (4 sec) with the correct and wrong responses, and histological confirmation of the sites of the electrodes in mPFC and vHPC (LFP: local field potential; mPFC: medial prefrontal cortex; vHPC: ventral hippocampus).

5. In the abstract I presume that the sentence "animals were cannulated" refers to electrodes implant; rephrase.

**Answer: Sorry for this mistake, we rephrased the sentence in the abstract.**

**#Abstract: In the 9th postnatal week, the tube test was started to determine the relative social rank in each cage. One month after living in the hierarchy, the we implanted electrodes in mPFC and vHPC.**

**References**

1. O'Neill PK, Gordon JA, Sigurdsson T. Theta oscillations in the medial prefrontal cortex are modulated by spatial working memory and synchronize with the hippocampus through its ventral subregion. Journal of Neuroscience. 2013 Aug 28;33(35):14211-24.

2. Navi FF, Heysieattalab S, Raoufy MR, Sabaghypour S, Nazari M, Nazari MA. Adaptive Closed-Loop Modulation of Cortical Theta Oscillations: Insights into the Neural Dynamics of Navigational Decision-Making. Brain Stimulation. 2024 Sep 12.
