# Supplementary material for: Social subordination is associated with better cognitive performance and higher theta coherence of the mPFC-vHPC circuit in male rats
Source: PLoS One. 2025 Apr 16;20(4):e0320952. doi: 10.1371/journal.pone.0320952 (PMC12002502; doi:10.1371/journal.pone.0320952)
Supplement: Attachment — Submitted filename: Respnse to Reviewer_ 1403_11_14.docx [file pone.0320952.s003.docx]

**#Journal Requirements**:

1. Please review your reference list to ensure that it is complete and correct. If you have cited papers that have been retracted, please include the rationale for doing so in the manuscript text, or remove these references and replace them with relevant current references. Any changes to the reference list should be mentioned in the rebuttal letter that accompanies your revised manuscript. If you need to cite a retracted article, indicate the article’s retracted status in the References list and also include a citation and full reference for the retraction notice.

**Answer: Thanks for your comment, we ensure all of the references are complete and correct.**

**#Reviewer2**

1. in the Abstract the sentence "By increasing task difficulty, theta rhythm coherency was reduced for all social ranks but subordinates maintained better task performance with less reduction of theta coherence." should be reviewed and avoid the word 'coherency': Authors can use synchrony (or synchronization).

**Answer:** **Thanks for your comment, the mentioned item has been corrected in the text.**

**#Abstract: Although theta rhythm synchronization was reduced in all social ranks by increasing task difficulty, the subordinates maintained better task performance and less reduction of theta coherence.**

2. Also do not report "for the first time or similar in the abstract.

**Answer: we are grateful for your comment, it has been corrected.**

**#Abstract:** **These findings underscore the association between social hierarchy and working memory performance within the mPFC-vHPC circuit, highlighting the influence of social rank on implicated circuit.**

3. A further revision of the text to enhance its sound/readability would be appreciated.

**Answer: we thank the esteemed reviewer for making this point, we tried to make it readable.**

**#Abstarct:** Social dominance hierarchy is considered an influential factor on cognitive performance. The spatial working memory (SWM) is inversely related to dominance status after the formation of social hierarchy. However, their neural underpinings are poorly understood. The medial prefrontal cortex (mPFC) and ventral hippocampus (vHPC) play pivotal roles in social hierarchy and SWM. To investigate the associations between social hierarchy and SWM and their neural circuit (mPFC-vHPC), we used twenty-one natal male Wistar rats after weaning (3 rats per cage, 7 cages in total). In the 9th postnatal week, the tube test was started to determine the relative social rank in each cage (dominant, middle-ranked, subordinate). One month after living in the hierarchy, we implanted electrodes in mPFC and vHPC. One week following recovery, the SWM test was performed using T-maze with two difficulty levels (30s and 5min delays between trials) while recording the local field potentials. The percentage of correct responses showed no significant difference among three different social groups. However, subordinates demonstrated significantly lower latency in reaching the goal arm, while middle-ranked rats exhibited the longest latency in 30s delay. Electrophysiological data revealed significantly higher theta correlation and coherence of the mPFC-vHPC circuit in subordinates. Although theta rhythm synchronization was reduced in all social ranks by increasing task difficulty, the subordinates maintained better task performance and less reduction of theta coherence. These findings underscore the association between social hierarchy and working memory performance within the mPFC-vHPC circuit, highlighting the influence of social rank on implicated circuit.
